# Data rescue of historical tables through semi-supervised table structure recognition

**DOI:** 10.1007/s10032-025-00562-6

**Published:** 2025-12-01

**Authors:** Loitongbam Gyanendro Singh, Stuart E. Middleton

**Affiliations:** School of Electronics and Computer Science, University of Southampton, Southampton, UK

**Keywords:** Tabular Structure Recognition, Semi-Supervised Learning, Historical Document Digitization, Data Annotation Techniques

## Abstract

This study uses a novel semi-supervised learning framework to explore Tabular Structure Recognition (TSR) for digitizing historical documents, specifically employing the CascadeTabNet model. TSR is crucial for transforming archival tabular data into digital formats, enhancing accessibility and analysis across various research fields. Challenges like physical degradation, inconsistent lighting, and non-standard handwriting hinder the generation of high-quality annotations of historical documents needed for effective model training. To address these issues, this research explores two research questions: (i) *Can a semi-supervised training approach reduce the need for expensive data annotations?* and (ii) *Does semi-supervised training improve model robustness?* We applied our methodology across three datasets: the GloSAT and ICDAR-2019 datasets based on historical documents, and the predominantly modern documents PubTabNet dataset. Our results indicate that semi-supervised learning substantially increases TSR accuracy and decreases dependency on extensive labelled datasets, providing a robust solution for large-scale digitization initiatives and contributing to the preservation and improved accessibility of historical data. All code from this paper is freely available on GitHub (https://github.com/stuartemiddleton/glosat_table_dataset).

## Introduction

Data rescue initiatives are pivotal in preserving and digitizing historical documents and climate records, bridging the gap between the analog past and the digital future. These endeavors are crucial for safeguarding cultural heritage, scientific development, and our understanding of past climate patterns [1–4]. By converting decaying physical formats into digital forms, data becomes more accessible, analyzable, and storable, ensuring its availability for future research, sharing, and archiving. This is particularly vital for climate data, where digitized historical weather records offer invaluable insights into past climatic changes and trends, aiding in refining current climate models and forecasts [5, 6]. Moreover, digitization addresses the gaps in climate records, facilitating more comprehensive and efficient modeling and analysis, and reinforcing the critical convergence of historical authenticity and technological advancement [6, 7].

A core challenge in this digitization process lies in the intricate and densely packed tabular layouts found in historical climate logbooks. These documents often feature thousands of table cells on a single scanned page, presenting considerable difficulties for existing digitization methods. Current state-of-the-art (SOTA) digitization models, primarily designed for modern tabular data such as receipts or datasets like PubTabNet, struggle to manage the unique complexities of historical tables [8, 9]. Unlike modern tables, historical documents are scarce, irregular in structure (see Appendix Figures 7, 8, 9), and densely populated with information (refer Table 2), often requiring specialized approaches to ensure accurate digitization.

To address these challenges, the digitization process can be modularized into two key steps: Table Structure Recognition (TSR) and Text Extraction, leveraging the transfer learning capabilities of existing SOTA models. This paper highlights the critical role of TSR in ensuring the accuracy and reliability of digitized content and overcoming the challenges posed by historical documents. TSR focuses on accurately detecting cell boundaries, table structures, and their alignment, enabling the subsequent Optical Character Recognition (OCR) step to extract text from individual cells [10, 11]. This modular approach facilitates the reliable recovery of historical tabular data, enabling the creation of high-quality datasets for climate modeling and analysis. However, the digitization process is further complicated by challenges such as the need for precise, high-quality annotations, tables with densely packed cells, uneven layouts, and non-linear handwriting that extends into adjacent cell regions [10, 12]. Therefore, developing a robust TSR model is an essential step toward overcoming these obstacles and advancing the digitization of historical climate records.

Traditional approaches for building TSR models have primarily depended on fully supervised learning techniques, requiring extensively annotated datasets to train models capable of recognizing tabular structures [13]. While effective in controlled settings, these methods struggle with the variability and degradation present in historical records. To increase accuracy, sophisticated preprocessing methods have been developed to boost picture quality, and intricate models that are more suited to manage the variety in handwriting and layout have been used. Some studies have also explored integrating domain-specific knowledge to refine model predictions, focusing on custom solutions tailored to specific types of archival documents [14, 15]. However, these solutions often demand substantial manual effort in preparing training data and fine-tuning models, which can be prohibitive for large-scale digitization projects.

**Fig. 1:**
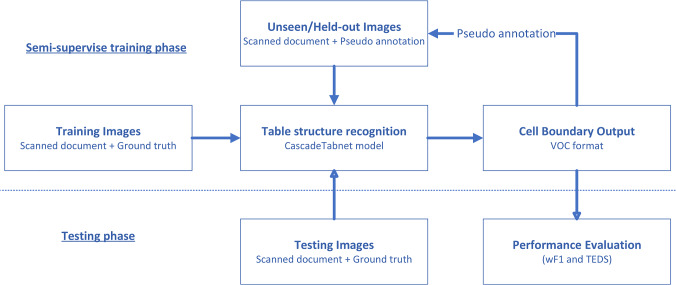
Semi-supervised learning framework for Tabular Structure Recognition, illustrating training and testing phases: during training, the model predicts table cell bounding boxes as pseudo annotations to iteratively improve with unlabelled data; during testing, the trained model’s outputs are evaluated using wF1 and TEDS metrics

Building on the abovementioned challenges, this study proposes a semi-supervised learning approach for TSR, leveraging SOTA models as an alternative to fully supervised methods. Figure 1 presents an overview pipeline of the semi-supervised learning approach. This approach is designed to address two key research questions: (i) *Can a semi-supervised training approach reduce the need for expensive data annotations?* and (ii) *Does semi-supervised training improve model robustness?* By combining both labeled and unlabeled data within a semi-supervised framework, this study aims to alleviate the burden of extensive data annotation while enhancing model accuracy and resilience to the variability and degradation of historical documents. This novel strategy represents a significant step toward scalable and efficient digitization of historical tabular data, aligning with the broader goal of creating reliable datasets for climate research and analysis.

To evaluate the efficiency of the semi-supervised learning process, the training set is divided into six partitions with varying proportions of labelled data. This enables a detailed analysis of how different amounts of labelled data impact model performance, particularly in scenarios with limited annotations. Rather than benchmarking all SOTA models [8, 16, 17], this study focuses on assessing the feasibility and effectiveness of a semi-supervised learning approach under such constraints. The CascadeTabNet model [18] was selected as the representative SOTA model for this study due to its strong performance in the ICDAR-2019 (Track B1) post-competition results [19]. Table 1 compares the semi-supervised training performance of CascadeTabNet [18] with the benchmark models such as UniTabNet [8] and TSR-Net [16], highlighting the justification for selecting CascadeTabNet to evaluate our proposed semi-supervised learning approach. Detailed experimental results for semi-supervised training are provided in Tables 5 and 6.

**Table 1:** Performance comparison of the proposed semi-supervised finetuned CascadeTabNet model with counterpart models like UniTabNet [8] and TSR-Net [16] (original results reported by the authors) on ICDAR-2019 (Track B1) and PubTabNet datasets

	ICDAR-19 (Track B1)		PubTabNet	
Model	wF1	TEDS	wF1	TEDS
UniTable [8]	–	–	–	0.965
TSR-Net [16]	–	–	**0.852**	0.956
ICDAR-2019 Competition [19]	0.485	–	–	–
CascadeTabNet (Semi-supervised)	**0.529**	**0.487**	$0.382^*$	${\textbf {0.975}}^*$

* The results for the PubTabnet dataset were finetuned with a subset of 6000 samples

By utilizing CascadeTabNet, this paper systematically investigates how a semi-supervised approach can improve model performance by combining a limited set of labeled data with a larger amount of unlabeled data. We evaluated TSR performance using two widely recognized evaluation metrics: the Weighted Average F1 (wF1) score [10, 15, 19, 20] and the Tree-Edit-Distance-based Similarity (TEDS) [17, 20]. These metrics provide a comprehensive framework for comparing semi-supervised and fully supervised learning outcomes, highlighting the potential benefits of our approach in reducing dependence on extensively annotated datasets. Ultimately, the semi-supervised learning approach offers a robust solution for large-scale digitization projects, significantly enhancing the accessibility and preservation of important historical documents.

In summary, the following are the main contributions of this study:

- Proposes a novel semi-supervised training approach, leveraging a pretrained table structure recognition model to reduce annotation requirements and enhance model robustness.
- Provides a comprehensive performance evaluation using wF1 and TEDS metrics to compare semi-supervised and fully supervised learning models.

## Related studies

Deep learning has dramatically transformed the field of table detection and structure recognition in document images. Traditionally reliant on heuristics and rule-based methods [21, 22], the field has recently pivoted to sophisticated deep learning techniques. Popular object detection models like Faster R-CNN [23], RetinaNet [24], and YOLO [25], alongside segmentation models such as TableNet [26], CascadeTabNet [18], have been adeptly adapted for TSR task. Additionally, transformer-based models like DiT (Document Image Transformer) [27] and TableFormer [28] have set new benchmarks by integrating advanced models like DETR [29] and Cascade-RCNN [30] for TSR pipelines. DiT enhances generalizability across diverse datasets through self-supervised pre-training on document images. In contrast, TableFormer delivers exceptional results by incorporating architectural advancements like the novel object detection decoder and transformer-based decoders. However, training these complex models requires a large annotated dataset to excel in learning complex table structures without manual feature engineering.

Existing large-scale datasets such as TableBank [31], PubTabNet [17], and FinTabNet [20] have already significantly advanced end-to-end table recognition research, particularly for a broad range of modern documents. Despite these advances, challenges remain in applying these techniques to historical documents, which often exhibit degraded quality, diverse layouts, and handwritten elements. For example, while CascadeTabNet demonstrates excellence in benchmarks like ICDAR 2019 [19] and GloSAT [32] datasets, its performance is less effective for the complex requirements of historical documents. This issue is addressed through specific adaptations for historical document analysis, such as the DIVA-DAF framework [14] and RobusTabNet approach [15], which offer tailored segmentation and classification but face difficulties with the variability in format and quality of historical documents. These frameworks underscore the necessity for more robust models and the development of comprehensive datasets that capture the complexity of historical tabular data more accurately.

Our research diverges from existing efforts by using semi-supervised learning to improve model performance with limited annotated data, specifically for historical document collections. This approach minimizes dependence on large labelled datasets and increases training efficiency by integrating both labelled and unlabelled data. This is particularly crucial for historical documents, where acquiring labelled examples is difficult and expensive. We focus on refining deep learning techniques to manage the varied table layouts and formats in historical and diverse document collections, highlighting our unique approach to improving data accessibility and preservation.

## Research methodologies

**Table 2:** Dataset distribution overview showing the number of samples, proportions of handwritten and typed documents, and the average number of table cells per image for GloSAT, ICDAR-2019 Track B1 (historical datasets), and PubTabNet (modern dataset)

Dataset	#Trainset	#Testset	Average cells/image	% of handwritten documents	% of typed documents
GloSAT	454	46	859.85	50%	50%
ICDAR-2019	600	150	355.97	88%	12%
PubTabNet	$6000^*$	9115	40.14	–	100%

* The original PubTabNet dataset was released with 510K training samples

**Table 3:** Data distribution of climate logbooks in the GloSAT dataset

Types of logbooks	Location	Year	Table layouts per logbook	# Labelled images	Average cells/image
Logbook1	India (MO)	1970	2	24	1971.667
Logbook2	India (NOAA)	1930	1	24	2197.429
Logbook3	Philippines	1900	2	24	740.292
Logbook4	Natal, Africa	1870	1	26	99.429
$\text {Logbook5}^\checkmark$	UK	1830-1930	2	97	208.959
$\text {Logbook6}^\checkmark$	UK and World	1900	8	93	622.793
$\text {Logbook7}^\checkmark$	Artics	1880	1	82	477.122
Logbook8	Ben Nevis, UK	1890	2	97	1511.247
$\text {Logbook9}^\checkmark$	Devon, UK	1890-1940	2	33	229.545

$^\checkmark$ The logbooks contain a mix of handwritten and typed text

### Datasets

This study employs semi-supervised learning to evaluate Tabular Structure Recognition (TSR) across three distinct datasets: the GloSAT dataset [32], which consists of historical climate logbooks dating back to the $18^{th}$ century; the ICDAR-2019 dataset [19], focused on historical documents; and the modern, document-centric PubTabNet dataset [17]. These datasets were carefully selected to assess the effectiveness of semi-supervised learning in recognizing and digitizing tabular data across both historical and contemporary contexts. Detailed information on the distribution and characteristics of these datasets is provided in Table 2. Additionally, Table 3 offers further insights into the types of logbooks used in the GloSAT dataset, including their sources, time periods, the mix of typed and handwritten information, and the average number of cells present per logbook.

To evaluate semi-supervised learning for TSR, we partition the training dataset into six segments, corresponding to different proportions of labelled data, ranging from 17% to 100%. This method helps assess the minimum amount of labelled data needed for effective training while utilizing unlabelled data to enhance the learning process. For example, in the 50% partition, half of the dataset is used for initial training, with the other half serving as unlabelled data for refinement. In smaller partitions (17% and 33%), held-out data from the 50% partition is used, while larger partitions (above 50%) incorporate additional held-out data. This incremental partitioning helps examine if smaller subsets of labelled data suffice for TSR under semi-supervised training. To ensure a fair comparison, the supervised baseline models are trained on the same labelled data partitions used in the semi-supervised experiments, excluding the held-out unlabelled data. The detailed distribution of these partitions is provided in Table 4.

To further investigate the effect of dataset size on table structure recognition performance, we created a representative subset from the large PubTabNet dataset, which contains 510,000 training samples. We randomly sampled 6,000 images to balance computational feasibility (approximately 120 hours of training) with robust evaluation. This random sampling preserved the original dataset’s distribution, ensuring the subset remained representative. The 6,000 samples were then evenly divided into six subsets, enabling direct and consistent comparisons with smaller benchmark datasets such as ICDAR (600 samples) and GloSAT (500 samples). This experimental design allows us to systematically evaluate how increasing annotation volume influences model training and performance.

### CascadeTabNet model

This study evaluates the effectiveness of a semi-supervised learning framework using the CascadeTabNet model, renowned for its performance in the ICDAR-2019 post-competition results, to enhance Tabular Structure Recognition (TSR) in historical documents. Focusing on CascadeTabNet, we explore the benefits of our semi-supervised approach that integrates a limited amount of labelled data with a larger pool of unlabelled data, a method especially beneficial in TSR, where labelled data are scarce and expensive. This approach is applied in three distinct datasets to demonstrate its potential to significantly improve model performance and navigate the unique challenges of tabular data recognition in historical documents.

The CascadeTabNet model employs a Cascade Mask R-CNN architecture [30] with a High-Resolution Network (HRNetV2p_W32) backbone [33] that extracts detailed, multi-scale features from input document images. These features are subsequently integrated using a feature pyramid network, and a region proposal mechanism then generates initial proposals for potential table locations. These proposals are refined through a feature alignment process that normalizes inputs of various sizes into consistent feature maps. As the process progresses, CascadeTabNet performs multiple detection stages, each responsible for dual predictions: one for the presence of a table or table cell and another for the precise coordinates of their bounding boxes. By adopting a class-agnostic approach to bounding box regression, CascadeTabNet minimizes computational demands while delivering robust performance. This makes it ideal for precisely recognizing tables and table structures across diverse document scenarios. The iterative refinement ensures accurate detection of both tables and their layout structures, maintaining consistent accuracy across different document types. The final output includes classifications of table regions and their precise coordinates, as well as the delineation of individual table cells.

**Table 4:** Distribution of training set partitions into six segments to evaluate semi-supervised learning performance. Held-out samples are treated as unlabelled data for semi-supervised training

Training Samples %	#Trainset	#Held-out	#Testset
GloSAT			
16.67%	74	223	46
33.33%	149	223	46
50.00%	223	223	46
66.67%	297	149	46
83.33%	372	74	46
100%	454	–	46
ICDAR-2019			
16.67%	100	300	150
33.33%	200	300	150
50.00%	300	300	150
66.67%	400	200	150
83.33%	500	100	150
100%	600	–	150
PubTabNet			
16.67%	1000	3000	9115
33.33%	2000	3000	9115
50.00%	3000	3000	9115
66.67%	4000	2000	9115
83.33%	5000	1000	9115
100%	6000	–	9115

### Semi-supervised weakly labelled training regime

This section presents our proposed semi-supervised weakly labelled training regime, aimed at improving table structure recognition by combining a limited amount of labelled data with a larger pool of unlabelled or weakly labelled samples. Rather than relying solely on expensive and time-consuming manual annotation, this approach uses the model’s own predictions on unlabelled data as weak annotations. These predictions are incorporated iteratively to retrain and refine the model, enhancing its ability to accurately reconstruct complete table layouts. We demonstrate this strategy using CascadeTabNet to maximize the utility of the data and reduce the dependency of the annotation.

CascadeTabNet dynamically predicts the number of table cells per image based on the image content and the region proposals generated during training. As detailed in Appendix Table 8, tuning parameters such as the number of output channels or classes strongly influence the computational demands and efficiency of the model. Increasing model complexity can improve detection accuracy, but also raises the risk of overfitting, particularly when training data are limited.

To address both computational constraints and the scarcity of fully labelled data, our framework adopts a heuristic centroid-based method to detect and infer missing cells, especially in the table body, where critical information is concentrated. After initial cell detection, each cell’s centroid is computed and organized into a grid structure that serves as a spatial reference for the table. The grid is then refined by merging centroids that lie closer to the average cell size, ensuring that each centroid corresponds to a unique cell location. From this refined grid, missing candidate cells are generated by extrapolating average cell dimensions, and only candidates aligned with detected centroids are retained. This process robustly reconstructs table layouts even when labels are incomplete or noisy.

Within the semi-supervised training loop, these predicted cell layouts act as weak annotations on unlabelled images, enabling iterative retraining to progressively improve model accuracy. In each iteration, the model is initialized with the weights from the most recently trained checkpoint, ensuring that learning builds upon the latest improvements^1^. By effectively combining limited fully labelled data with weakly labelled unlabelled examples, this approach reduces reliance on labor-intensive manual labeling and boosts robustness. This is particularly important in domains such as historical document analysis, where large-scale, high-quality annotations are challenging and costly to obtain. Given the iterative nature of this training regime, each iteration requires approximately 120 hours to complete on a system equipped with an Intel Xeon processor (3.6 GHz), 64 GB DDR4 memory, and an NVIDIA Quadro RTX 8000 GPU with 48GB of memory.

**Fig. 2:**
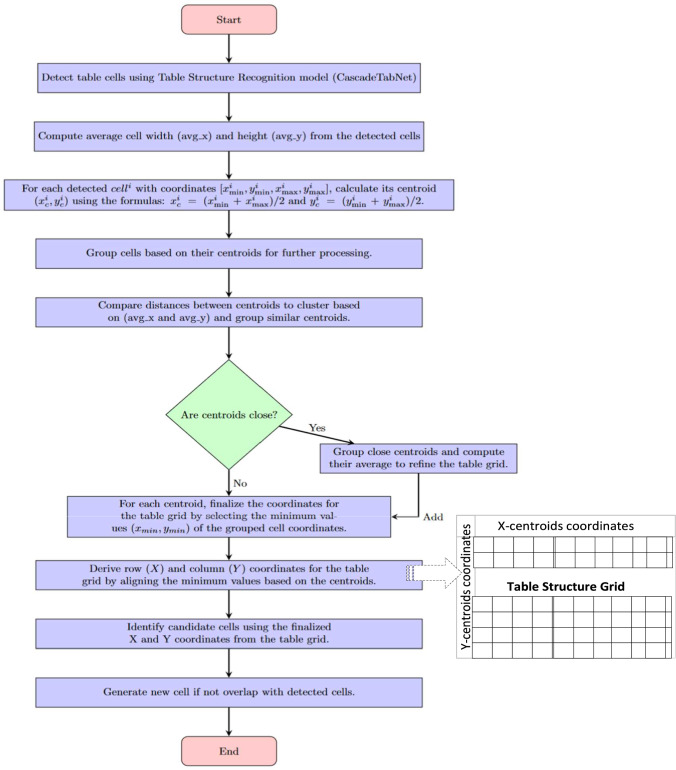
Flowchart illustrating the step-by-step process for detecting and generating missing table cells using centroid-based grid refinement during semi-supervised training

Figure 2 provides a detailed flowchart of the reconstruction and training workflow, while Appendix Figures 7, 8, and 9 show representative output examples. Our implementation of CascadeTabNet and the semi-supervised training algorithm is publicly available on GitHub^2^, facilitating easy reproduction and extension of this methodology to other table structure recognition models.

### Evaluation metrics

Our study evaluates Table Structure Recognition (TSR) performance using two key metrics: the Weighted Average F1 (wF1) score, aligned with the cTDaR-19 competition standards [19], which emphasizes the precision and recall of the model across varying IoU thresholds, and Tree Edit Distance-Based Similarity (TEDS) [17], which measures the precision of the model in aligning the predicted table structures with the actual, focusing on both layout and content. These metrics offer a detailed assessment of the TSR models, capturing their effectiveness in document analysis with a comprehensive view of detection accuracy and structural fidelity.

1 $$\begin{aligned} \text {wF1} = \sum _{i} w_i \cdot \frac{2 \cdot \text {Precision}_i \cdot \text {Recall}_i}{\text {Precision}_i + \text {Recall}_i} \end{aligned}$$

Here, $w_i$ is the weight for the Intersection over Union (IoU) threshold *i*, and ${Precision}_i$ and ${Recall}_i$ are the precision and recall at the $i^{th}$ IoU threshold. The IoU thresholds, set at 0.6, 0.7, 0.8, and 0.9 as per the cTDaR-19 competition standards [19], define the minimum overlap required between predicted and ground truth table cells for a prediction to be considered correct. Using these thresholds ensures a comprehensive evaluation by balancing precision and recall under varying levels of overlap, capturing the model’s performance in both lenient and strict conditions.

2 $$\begin{aligned} \text {TEDS}(T_a, T_b) = 1 - \frac{\text {EditDist}(T_a, T_b)}{\max (\Vert T_a\Vert , \Vert T_b\Vert )} \end{aligned}$$

where $T_a$ and $T_b$ represent the tree structures of the actual and predicted tables. EditDist$(T_a, T_b)$ is the tree-edit distance between these trees, and $\Vert T_a\Vert$ is the number of nodes in a tree $T_a$.

## Results and discussion

**Table 5:** Performance comparison (wF1 scores) of supervised and semi-supervised TSR models across varying data partitions ranging from 16% to 100% labelled data. The 83% semi-supervised models outperformed the 100% supervised models, showing improvements and robustness of the semi-supervised training strategy

		Data split percentage					
Approach	Dataset	16.67%	33.33%	50%	66.67%	83.33%	100%
	GloSAT	0.313	0.362	0.440	0.467	0.478	0.486
Supervised	ICDAR-2019	0.453	0.455	0.465	0.474	0.475	0.485
	PubTabNet	0.312	0.331	0.355	0.349	0.362	0.367
	GloSAT	0.297	0.383	0.472	0.473	**0.495**	–
Semi-supervised	ICDAR-2019	0.444	0.477	0.475	0.497	**0.529**	–
	PubTabNet	0.301	0.326	0.368	0.368	**0.382**	–

**Table 6:** Performance comparison (TEDS scores) of supervised and semi-supervised TSR models across varying data partitions ranging from 16% to 100% labelled data

		Data split percentage					
Approach	Dataset	16.67%	33.33%	50%	66.67%	83.33%	100%
	GloSAT	0.868	0.865	0.861	0.865	0.875	0.880
Supervised	ICDAR-2019	0.378	0.406	0.407	0.417	0.438	0.470
	PubTabNet	0.926	0.929	0.934	0.939	0.950	0.954
	GloSAT	0.862	0.874	0.880	0.883	**0.890**	–
Semi-supervised	ICDAR-2019	0.370	0.411	0.413	0.425	**0.487**	–
	PubTabNet	0.910	0.913	0.952	0.953	**0.975**	–

### Minimum labelled data required for effective training

Tables 5 and 6 comprehensively analyze both supervised and semi-supervised training models for Tabular Structure Recognition (TSR) across three datasets using wF1-score and TEDS metrics, particularly focusing on determining the minimal amount of labelled data required for effective training. The analysis reveals that in partitions below 50%, semi-supervised methods initially lag behind supervised approaches, especially for the PubTabNet dataset. However, as the labelled data percentage in semi-supervised training increases, these models show marked performance improvements. Notably, by the 50% partition, semi-supervised models outperform their fully supervised counterparts across all datasets. When the labelled data reaches 83%, the semi-supervised models outperformed the fully supervised models trained with 100% training data, demonstrating the improvements and robustness in utilizing the strengths of semi-supervised learning to achieve high performance with fewer labelled examples.

Additionally, the observed performance enhancements in semi-supervised learning highlight the model’s increasing efficiency as more labelled data becomes available. This supports the core objective of identifying the minimal labelled data threshold necessary for effective training. Integrating unlabelled data with labelled data helps the model generalize more effectively, showcasing a critical strategy for optimizing resource use in training setups. The analysis also underscores the practical benefits of semi-supervised learning, particularly when acquiring extensive labelled data is challenging. This approach reduces dependency on large labelled datasets. It improves model performance through better generalization and adaptability to variations in data, aligning closely to maximize training efficiency with minimal labelled data.

Our findings reveal a discrepancy between high TEDS scores and low wF1 scores for table structure recognition, particularly in the GloSAT and PubTabNet datasets. TEDS, which evaluates the overall similarity of the table’s tree representation, is more forgiving of minor structural errors, resulting in higher scores. In contrast, the wF1 score, which measures precision and recall, penalizes even small deviations from the ground truth. This contrast highlights the model’s strength in capturing the general table structure but also its challenges in accurately localizing and classifying individual table cells.

**Fig. 3:**
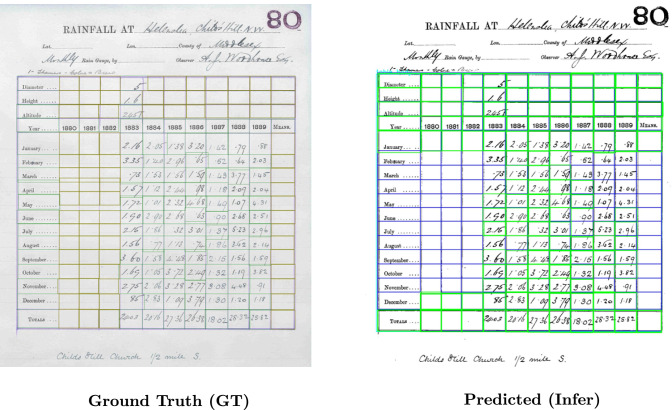
Comparison of Ground Truth (GT) and Predicted (Infer) table structures. The GT annotations accurately align with text regions, whereas the Predicted structure shows slight misalignments, resulting in a wF1 score of 0.847 and a TEDS score of 0.99. The cells highlighted in blue represent the predictions made by CascadeTabNet, while those in green indicate the newly generated cells derived from the coordinates of the blue-highlighted cells

The stricter evaluation of wF1 amplifies the impact of misalignments or overlaps, even when these errors do not significantly alter the overall structure as assessed by TEDS. For instance, in the example shown in Figure 3, the ground truth annotations are highly precise, aligning perfectly with text region boundaries, while the model predictions exhibit slight misalignments, leading to a wF1 score of 0.847 and a TEDS score of 0.99. These minor discrepancies reduce the IoU and, subsequently, the wF1 score, despite the model’s ability to accurately capture the table layout.

For the ICDAR-2019 dataset, which features diverse table layouts, the model faces additional challenges in recognizing densely packed table cells. Although our heuristic approach improves the detection of some missing adjacent cells, its effectiveness is limited due to the small size of the training set for specific tabular layouts. As a result, the TEDS and wF1 scores for this dataset are relatively closer. Nevertheless, our model achieved a significant milestone by outperforming the competition’s best performer, achieving a higher wF1 score of 0.53 compared to 0.49^3^. This result underscores the robustness of our semi-supervised learning strategy, demonstrating its potential to advance table structure recognition.

The contrast between TEDS and wF1 scores not only highlights the model’s performance differences across datasets but also underscores the importance of stricter evaluation metrics like wF1 for assessing real-world applicability. The stricter nature of the wF1 metric is not merely a limitation but a critical feature for evaluating real-world applications. Stricter metrics like wF1 are particularly valuable for downstream tasks, such as text extraction, which depend on precisely localized text regions for accurate digitization. While our model excels in capturing the overall table structure, its limitations in fine-grained localization and classification accuracy highlight the need for further improvements. Addressing these challenges is crucial for enabling high-quality digitization, especially in dense and complex layouts commonly found in historical documents, where precision is paramount for achieving accurate text extraction.

### Stability analysis of TSR model across data partitions

To further evaluate the robustness of our semi-supervised learning model for the Tabular Structure Recognition (TSR) task, we conducted a detailed stability analysis using shuffled partitions of the ICDAR-2019 dataset. This analysis involved dividing the dataset into three distinct sets. Each was randomly shuffled to ensure no systematic biases influenced the model’s learning and performance outcomes.

**Fig. 4:**
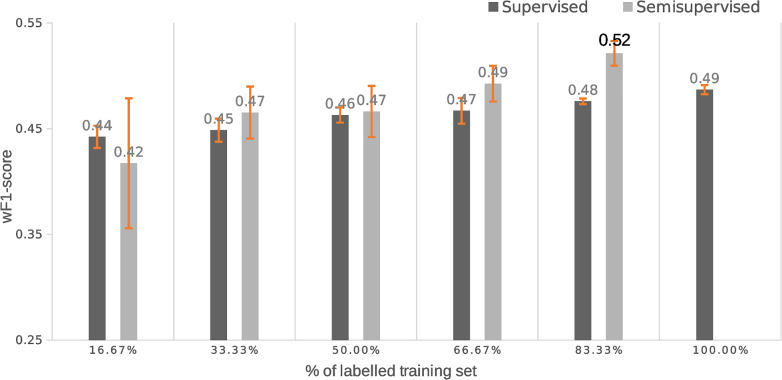
Performance of the TSR model (wF1-scores) across three shuffled partitions of the ICDAR-2019 dataset, illustrated with mean values and error bars to assess consistency and error biases. Detailed distribution in Appendix Table 9

The objective was to ascertain whether the model’s performance would remain consistent across various data partitions, a crucial factor for practical applications where data variability is inevitable. Figure 4 presents a bar chart that visualizes the mean performance of the TSR model on three shuffled sets from the ICDAR-2019 dataset, complemented by error bars that depict the consistency and potential error biases. Each bar indicates the model’s performance on a distinct partition, highlighting the stability of the learning model under various training conditions. The results indicated a high consistency in model performance, with an error margin as narrow as 0.004 standard deviations. This low variance affirms the model’s effectiveness in handling different data configurations and its reliability for operational use. The consistent performance across shuffled data partitions reinforces the robustness and reliability of our semi-supervised learning approach, suggesting minimal error biases when handling diverse sets within the same dataset. This finding supports the practical application of this model in environments where dealing with data variability is a common challenge.

**Table 7:** Data distribution of climate logbooks in GloSAT dataset used for semi-supervised training. The unlabelled images are considered for semi-supervised enhancement of the TSR model on the underperformed logbooks

Types of logbooks	# Layouts per logbook	#Labelled images	Average cells/image	#Unlabelled images	# Semi-supervised annotation
Logbook1	2	24	1971.667	276	73
Logbook2	1	24	2197.429	380	70
Logbook3	2	24	740.292	6077	67
Logbook4	1	26	99.429	46	–
Logbook5	2	97	208.959	–	–
Logbook6	8	93	622.793	1330	414
Logbook7	1	82	477.122	–	–
Logbook8	2	97	1511.247	–	–
Logbook9	2	33	229.545	–	–
Total	21	500	–	8109	624

### Exploring unseen data robustness through iterative semi-supervised learning

The semi-supervised TSR model, trained with 83% labelled data from the GloSAT dataset, demonstrates inconsistent performance across various logbooks in the test set, identifying the need for more precise annotations to improve predictions. The limited availability of ground-truth data complicates efforts to enhance model accuracy. To address this, we employed the semi-supervised model to annotate unannotated images from logbooks where the model scored below 50% wF1-scores. We gathered additional images from four logbooks, totaling 8109 unannotated images as outlined in Table 7. After meticulously reviewing the most accurately predicted images, we weakly labelled 634 of them and added them to the training set, thereby augmenting the existing fully labelled data. This method enriches the dataset and elevates model performance in areas where it previously fell short.

**Fig. 5:**
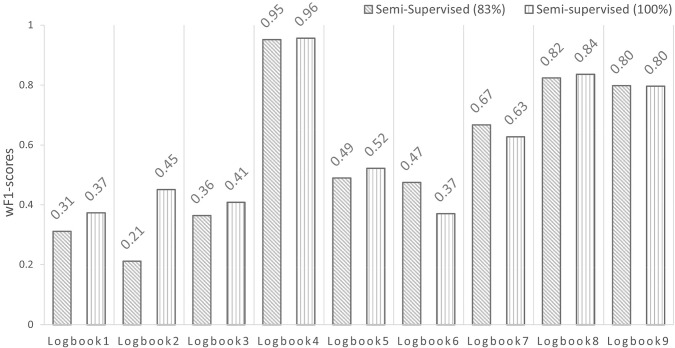
Performance comparison of semi-supervised TSR models, utilizing weakly labelled with 83% and 100% labelled training sets, across various climate logbooks in the GloSAT dataset

Figure 5 illustrates a performance comparison of semi-supervised models trained with 83% and 100% of the training set, using weakly labelled across various climate logbooks in the GloSAT dataset. Notably, the model trained with 100% of the training set demonstrates enhanced performance, particularly in logbooks where the model trained with 83% had previously underperformed. However, an exception is noted in *Logbook6*, where performance varies. This variation is due to the diverse tabular layouts in Logbook6 (i.e., 8 layouts); the model excels for layouts with more training examples but faces challenges with other layouts when less weakly labelled examples are included in the training. This iterative process of annotating and retraining, which may include revisiting previously excluded unannotated images, is designed to expand the training base continually. This strategy not only utilizes the potential of unlabelled images but also systematically refines the training set with high-quality annotations, thereby progressively enhancing the overall performance of the TSR model.

**Fig. 6:**
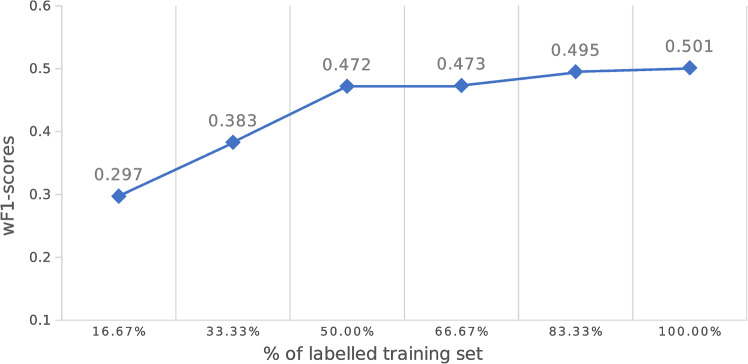
Enhancement in semi-supervised model performance with additional GloSAT unlabelled data

Figure 6 shows how incorporating more weakly labelled training data increased the semi-supervised model’s performance from a wF1 score of 0.297 to 0.501, exceeding that of a fully supervised model of 0.486 wF1-score. These gains are attributed to improved generalization, reduced overfitting, and enhanced feature representation on inspection. This demonstrates the practical benefits of semi-supervised learning in environments where extensive, fully labelled datasets are unavailable. Significant enhancements demonstrated across multiple training iterations underscore the model’s ability to effectively use generated data for ongoing improvements. This iterative training process showcases the potential of semi-supervised learning to efficiently manage datasets with limited or no ground-truth annotations, a crucial advantage in scenarios where acquiring labelled data is challenging.

## Conclusion

This study demonstrated the efficacy of semi-supervised learning approaches for Table Structure Recognition (TSR) in digitizing historical documents. By evaluating performance across three datasets - GloSAT, ICDAR-2019, and PubTabNet, we demonstrated how semi-supervised models could minimize reliance on extensively labelled datasets, addressing a major challenge in historical document analysis. A key observation was the variance between high Tree Edit Distance Scores (TEDS) and lower weighted average F1 (wF1) scores. This indicates that while the models adeptly captured the general layout of tables (high TEDS), they struggled with accurately localizing individual table cells (lower wF1), a difficulty exacerbated by the complex layouts and deteriorated quality of historical documents. However, the inclusion of semi-supervised training using weakly labelled training data and iterative re-training significantly improved accuracy. This iterative semi-supervised approach enabled the models to surpass the performance of their fully supervised counterparts trained only on limited labelled data. The implications of these findings are two-fold: (a) Semi-supervised learning emerges as a promising strategy for overcoming the scarcity of labelled data in historical document analysis, a field where obtaining extensive manual annotations is challenging and expensive; (b) Despite difficulties in precise cell localization for complex table layouts, the strong performance in capturing overall table structures suggests that semi-supervised techniques could facilitate rapid digitization of tabular data from historical sources.

**Table 8:** Parameters for training CascadeTabNet Model

Description	Value
Input image size (height x width)	1024x1024
Backbone model used for feature extraction	ResNet-50
Number of output channels in the last layer of the backbone	256
Number of input channels	256
Number of fully connected (FC) layers	2
Number of output channels for each FC layer	1024
Number of stages in the cascade	3
Region Proposal Network (RPN) output threshold	2000
RPN minimum positive IoU threshold	0.3
Number of object classes (table or background)	2
Optimizer	SGD
Learning rate of the optimizer	0.005
# Epochs (early stopping strategy)	600
Loss function for classification	Cross Entropy
Loss function for bounding box regression	Smooth L1

**Table 9:** Performance of the TSR model (wF1-scores) across three shuffled partitions of the ICDAR-2019 dataset

	Data split percentage					
Dataset	16.67%	33.33%	50%	66.67%	83.33%	100%
	Supervised approach					
Set 1	0.453	0.455	0.465	0.474	0.475	0.485
Set 2	0.442	0.436	0.455	0.453	0.474	0.484
Set 3	0.432	0.455	0.469	0.474	0.479	0.492
Mean	0.442 (± 0.011)	0.449 (± 0.011)	0.463 (± 0.007)	0.467 (± 0.012)	0.476 (± 0.002)	0.487 (± 0.004)
	Semi-supervised approach					
Set 1	0.444	0.477	0.475	0.497	0.529	–
Set 2	0.347	0.437	0.439	0.474	0.527	–
Set 3	0.461	0.482	0.485	0.507	0.508	–
Mean	0.417 (± 0.062)	0.465 (± 0.025)	0.466 (± 0.024)	0.493 (± 0.017)	0.521 (± 0.012)	–

## Limitations and Future work

While this study demonstrates the practical utility of semi-supervised learning for table structure recognition (TSR) in low-resource scenarios, it also presents several limitations that highlight opportunities for future research and development. First, this work does not propose novel architectural innovations; instead, it focuses on evaluating the effectiveness of applying a semi-supervised paradigm to an existing high-performing model, CascadeTabNet. This was a deliberate choice to isolate and understand the value of leveraging unlabelled data in a controlled and computationally feasible setting. Nevertheless, broader insights could be obtained by adapting this semi-supervised framework to other table recognition models with varying design principles. Second, the significant computational demands of training large-scale models pose practical limitations. Each training cycle, whether semi-supervised or supervised, requires approximately 120 hours on a high-performance system equipped with an Intel Xeon processor (3.6 GHz), 64 GB RAM, and an NVIDIA Quadro RTX 8000 GPU with 48GB memory. These constraints limited the scope of our experimental variations and precluded the use of large-scale ablations, ensemble configurations, or more frequent retraining schedules. Lastly, although the centroid-based heuristics used in this work effectively supplement weak annotations, more advanced techniques such as confidence-aware self-training, pseudo-label filtering, and uncertainty-based selection mechanisms could further improve weak supervision quality and overall model robustness.

In future work, we aim to enhance the capabilities of semi-supervised learning models for Table Structure Recognition (TSR) by focusing on three key strategies. First, we will improve weak labelling pipelines, particularly through enhanced synthetic data generation techniques to compensate for the shortage of annotated historical documents. Second, we intend to advance our machine learning framework by incorporating transfer learning and domain adaptation, allowing models to generalize more effectively across different document types, languages, and layouts. Lastly, we will foster cross-disciplinary collaborations with researchers in digital humanities, archival science, and computer science to refine and extend the applicability of our TSR methods for broader scholarly use. These future directions are designed to optimize semi-supervised TSR systems for real-world, domain-specific, and historically significant document collections.

## Figures and Tables

**Fig. 7:**
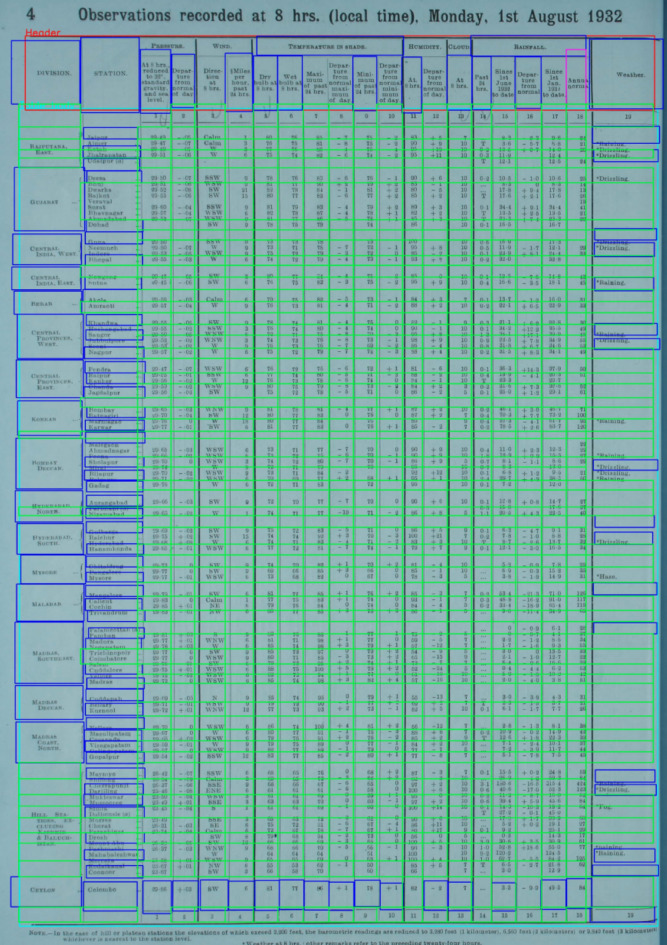
An example of semi-supervised weak labelling to generate missing cells for an unseen *Logbook2* image. The cells highlighted in blue represent the predictions made by CascadeTabnet, while those in green denote the newly generated cells derived from the coordinates of the blue-highlighted cells

**Fig. 8:**
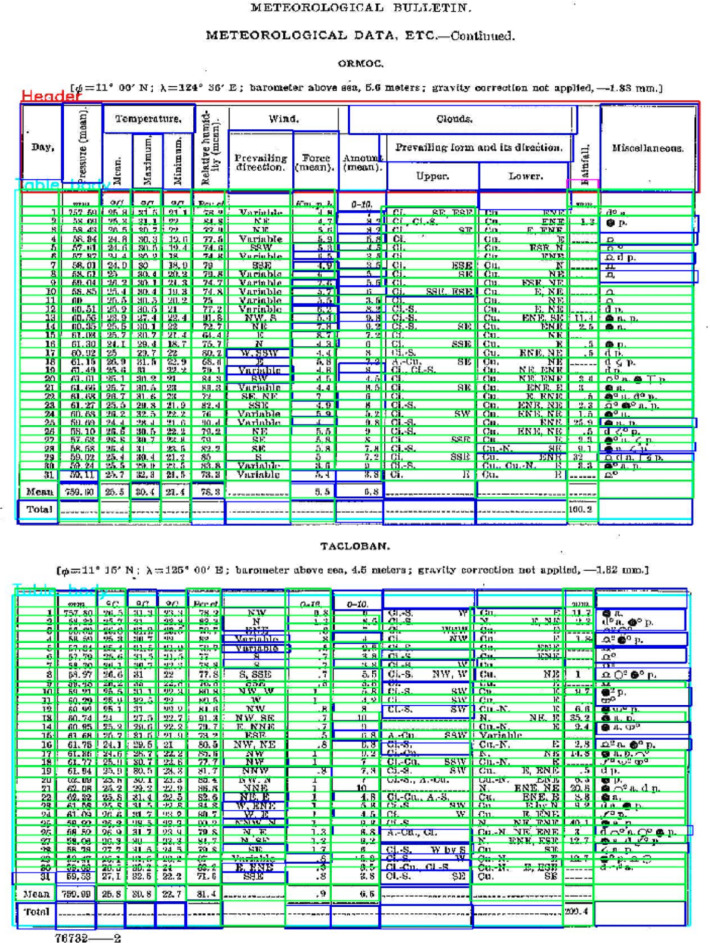
An example of semi-supervised weak labelling to generate missing cells for an unseen *Logbook3* image. The cells highlighted in blue represent the predictions made by CascadeTabnet, while those in green denote the newly generated cells derived from the coordinates of the blue-highlighted cells

**Fig. 9:**
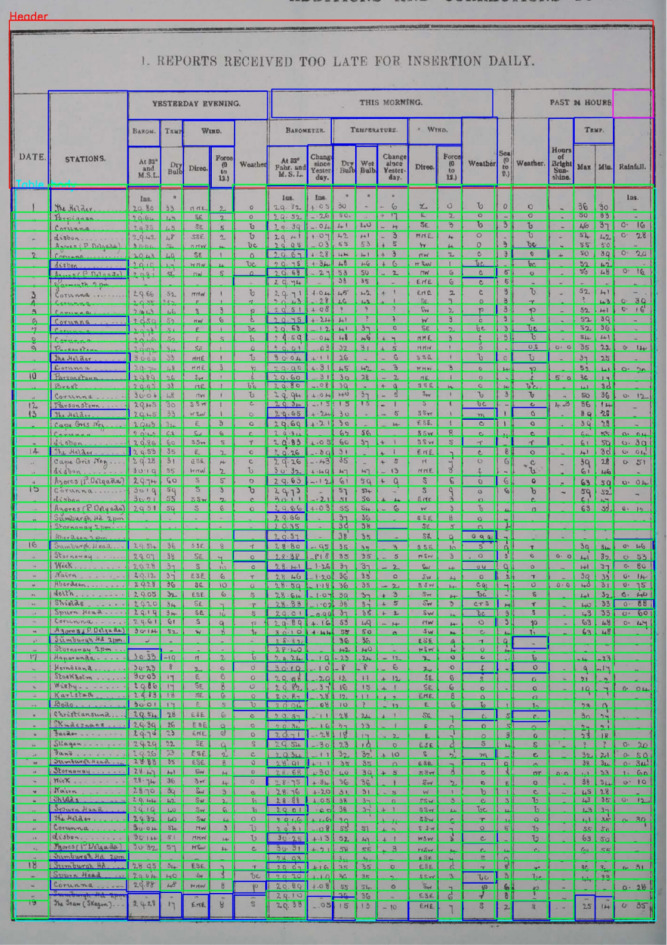
An example of semi-supervised weak labelling to generate missing cells for an unseen *Logbook6* image. The cells highlighted in blue represent the predictions made by CascadeTabnet, while those in green denote the newly generated cells derived from the coordinates of the blue-highlighted cells

## Data Availability

No datasets were generated or analysed during the current study.

## Appendix

Tables 9, 8 and Figures 7, 8, 9.

## Footnotes

1 This also applies to supervised training iterations.

2 https://github.com/stuartemiddleton/glosat_table_dataset

3 https://cndplab-founder.github.io/cTDaR2019/results.html.
